# Stemming the Shadow Pandemic: Integrating Sociolegal Services in Contact Tracing and Beyond

**DOI:** 10.1017/jme.2023.13

**Published:** 2022

**Authors:** Medha D. Makhlouf

**Affiliations:** 1. PENNSYLVANIA STATE UNIVERSITY, DICKINSON SCHOOL OF LAW, CARLISLE, PA, USA

**Keywords:** Health Equity, Covid-19, Poverty, Public Health, Medical-Legal Partnership

## Abstract

The COVID-19 pandemic has shed light on the challenges of complying with public health guidance to isolate or quarantine without access to adequate income, housing, food, and other resources. When people cannot safely isolate or quarantine during an outbreak of infectious disease, a critical public health strategy fails. This article proposes integrating sociolegal needs screening and services into contact tracing as a way to mitigate public health harms and pandemic-related health inequities.

Significant reductions in COVID-19 cases, hospitalizations, and deaths in the United States since the pandemic began have allowed many of us to imagine its end.^1^ However, the emergence of new variants means that traditional public health measures — including vaccination, testing, contact tracing, masking, quarantine, and isolation — remain an important component of the battle against COVID-19.^2^ Although the Omicron variants, which are highly transmissible and currently dominant, appear to cause relatively mild illness in most cases in the United States, it is possible that future variants will be just as transmissible but more deadly.^3^ Therefore, it is wise to review the measures that successfully mitigated public health harms during acute phases of the pandemic and create systems to support rapid implementation of those measures in the future.

This Commentary discusses lessons learned from contact tracing protocols during the COVID-19 pandemic. In general, these protocols rely on people’s ability to isolate or quarantine without direct support such as housing, income replacement, and food. However, many people in the United States are in financially precarious situations, owing both to acute changes caused by the pandemic (loss of employment) and to deep-rooted structural factors (discrimination, poverty). If they cannot afford to isolate or quarantine, this critical public health strategy to defeat the pandemic fails. I argue that integrating sociolegal needs screening and services into contact tracing is a promising strategy for mitigating public health harms and pandemic-related health inequities because it provides people with the resources they need to safely isolate or quarantine and builds trust in the public health system. This evidence supports broader integration of health care, public health, and human and legal services as preparation for the next COVID variant or viral pandemic.

## I. U.S. Public Health Authorities Rely on People to Comply with Isolation and Quarantine Instructions

Contact tracing is the chief public health measure for containing outbreaks of emerging, directly transmitted infectious disease.^4^ The standard formulation of the strategy for breaking the chain of transmission during such outbreaks is “test, trace, isolate.”^5^ Recent evidence suggests that of the three steps in the “test, trace, isolate,” process, isolation is the most important for interrupting the spread of the virus.^6^ When a person tests positive for COVID-19, contact tracers from a public health agency reach out to them to advise them to isolate and to determine their contacts who may be at risk of exposure.^7^ In the COVID-19 context, “close contacts” are then notified of the possible exposure and provided information about symptoms, testing resources, and quarantine guidelines.^8^ Symptomatic close contacts are advised to get tested and isolate.^9^ Before vaccines had been developed and were widely available, asymptomatic close contacts were advised to quarantine for 14 days from their last exposure.^10^ Under the most recently updated guidance issued by the U.S. Centers for Disease Control and Prevention (CDC), issued in March 2022, close contacts who are not up to date on COVID-19 vaccines are advised to quarantine for five days, while those who are up to date or who had confirmed COVID-19 within the last 90 days are not required to quarantine.^11^ Compared with other infectious diseases, contact tracing for COVID-19 is especially important and challenging because the virus is easily transmitted (through aerosol particles and respiratory droplets) by pre-symptomatic or asymptomatic people who have been infected.^12^

The success of efforts to improve the U.S. contact tracing system, such as those described in this commentary, depends on the existence of a system that is functioning effectively. Reforms not only *can* but *should* occur alongside efforts to improve implementation of the existing model.

Ensuring that people can safely isolate or quarantine is crucial to the success of contact tracing.^13^ In order to safely quarantine or isolate, people typically need safe and secure housing, a private bedroom and bathroom, access to sufficient amounts of nutritious food and clean water, uninterrupted electricity and gas utilities, access to laundry services, reliable telephone service, affordable health insurance with a reasonable actuarial value, a relationship with a primary care provider, over-the-counter medication, personal protective equipment (PPE), and cleaning supplies. Some may also need access to private transportation, childcare, the ability to continue earning income or wage replacement, and protection from termination from employment.

Although contact tracing seems simple in theory, it is quite complex and requires adequate resources to succeed.^14^ Results from studies of the effectiveness of contact tracing during the COVID-19 pandemic are limited due to the unavailability of data, and vary significantly.^15^ Notably, several East Asian countries — including South Korea, Vietnam, Japan, and Taiwan — successfully mounted contact tracing efforts early in the pandemic that helped to contain outbreaks.^16^ The reasons that so many countries, including the United States, failed to do so are “complex and systemic” but generally come down to underinvestment in public health and a lack of receptiveness to contact tracing due to distrust of public health authorities.^17^ Although the CDC no longer recommends universal contact tracing for COVID-19, contact tracing remains an important part of the toolkit for protecting people in high-risk settings and may be adopted more broadly in response to future variants.^18^ Therefore, it is premature to dismiss the potential utility of strengthening U.S. infrastructure for contact tracing as part of the strategy to mitigate harm during the COVID-19 pandemic or future epidemics.^19^ The success of efforts to improve the U.S. contact tracing system, such as those described in this article, depends on a the existence of a system that is functioning effectively. Reforms not only *can* but *should* occur alongside efforts to improve implementation of the existing model.^20^

## II. Failing to Address Resource Barriers to Isolation and Quarantine Undermines a Critical Public Health Strategy

When it is unsafe or impossible for people to safely isolate or quarantine, they have two bad options: (1) Disregard the instructions, which undermines a critical public health strategy, increasing the spread of disease and prolonging the pandemic; or (2) Comply with the instructions at great personal expense, possibly creating health risks for themselves or their household members, and exacerbating financial security. These harms will disproportionately fall on people who are already socioeconomically vulnerable and likely to be affected by health inequities.

The COVID-19 pandemic has shed light on the structural inequities creating financial and legal insecurity for many people living in the United States. Socioeconomic factors are having an outsized impact on morbidity and mortality from all causes during the pandemic.^21^ During every disaster, it is inevitably the communities that were marginalized and vulnerable prior to the disaster that suffer the most from it.^22^ Health care administrators have referred to the underlying social conditions that have influenced health outcomes during the pandemic as a “shadow pandemic.”^23^ The shadow pandemic is related to the social determinants of health, “the conditions in which people are born, grow, work, live, and age, and the wider set of forces and systems shaping the conditions of daily life.”^24^ Poor conditions shape health outcomes over time, sometimes through “long and complex pathways.”^25^ During disasters, however, the pathways connecting socioeconomic factors and health outcomes “become short and direct.”^26^ Structural inequities across several areas — including income, housing, food, and education — not only increase the risk of exposure for socioeconomically vulnerable groups, they also complicate their ability to safely isolate or quarantine when they or their close contacts test positive for COVID-19.^27^

These structural inequities have manifested as alarming racial and ethnic disparities in COVID-19 morbidity and mortality. Minoritized groups and households with the fewest resources are “disproportionately likely to hold jobs that require them to work outside the home; to lack stable, safe homes in which to shelter; to have limited access to affordable care; and to be affected by diseases of poverty such as chronic respiratory illnesses and diabetes that also increase risk for severe COVID-19 disease.”^28^ For Black and Latinx people, discrimination in health care adds to the risk.^29^ Altogether, unmet social and legal needs have contributed to the disparate impact of COVID-19 on minoritized communities during this global health emergency.

## III. Integrating Sociolegal Needs Screening and Services with Contact Tracing Helps to Ensure its Success

Integrating sociolegal needs screening and services into contact tracing is a promising strategy for mitigating public health harms and pandemic-related health disparities because it provides people with the resources they need to safely isolate or quarantine. Uniform integration of public health, health care, and human and legal services in contact tracing can help set the stage for more wide-reaching cross-sector collaborations in the interest of health justice. Addressing problems rooted in health inequity is the best preparation for the next COVID variant or viral pandemic.

From the earliest days of the COVID-19 pandemic, scholars have urged health care systems to maintain and even expand efforts to address the unmet social needs of all patients they encounter.^30^ Such efforts are driven by the understanding that structural inequities are the root causes of health inequities.^31^ Legal and policy advocacy can play an important role in addressing unmet social needs on the individual, institutional, and structural levels.^32^ Therefore, screening for and addressing unmet sociolegal needs could make a difference in the success of contact tracing efforts and help ameliorate the disproportionate impact of COVID-19 on racial and ethnic minorities.^33^

Integrating resource screening and supports in contact tracing is not a new idea, but it is not universally done.^34^ One model potentially worthy of emulation is Massachusetts’ state-funded Community Tracing Collaborative (CTC), which was established in April 2020 and aimed to provide all people who tested positive and their close contacts with the resources they need to isolate or quarantine.^35^ In July 2020, the CTC estimated that between 10-15% of people contacted requested assistance with meeting basic needs, including, most commonly, food, medicine, masks, cleaning supplies, and income support due to ineligibility for unemployment or rental assistance.^36^

Another program, the Test-to-Care Model, provided support to low-income Latinx residents of the Mission District in San Francisco who tested positive for COVID-19 in order to enable them to safely isolate.^37^ The Test-to-Care Model was a three-week demonstration project backed by university research funding.^38^ To address barriers relating to “environmental context and resources” during the isolation period, Community Health Workers (CHWs) delivered two weeks’ worth of groceries, PPE, cleaning supplies, hygiene products, over-the-counter medication, and information about enrolling in health insurance and establishing a relationship with a primary care provider.^39^ Upon completion of the isolation period, participants received an “exit package” consisting of face masks, vouchers to purchase groceries, and information about community resources, such as free testing sites.^40^ One of the key features of the program was that it provided *ongoing* screening, resource/service provision, and emotional support to participants during the isolation period.^41^ The results of this study were a catalyst for policy change that increased resources for people in San Francisco who tested positive for COVID-19: a low-barrier, city-funded “Right to Recover Program,” which provides wage replacement during the isolation and quarantine periods.^42^

Even in jurisdictions that have integrated social needs screening into contact tracing protocols, it is less common to see the integration of legal needs screening and legal services. Screening for legal needs and providing legal services to those with unmet needs can play an important role in permitting people to safely quarantine or isolate safely by, for example, advising people of their eligibility for health-supporting public benefits and other legal protections, such as eviction and utility shutoff moratoria, and appealing denials, terminations, or reductions of benefits. Lawyers can also help efforts to advocate for public investment in new forms of social assistance to meet emerging needs — such as income supports separate from unemployment, disability, and other cash assistance programs — and new legal protections. These are legal strategies for leveraging the law to improve the health and wellbeing of people with few resources. Although it is unclear if the Massachusetts CTC included a legal needs screening, news coverage of the program described how skilled resource coordinators identified unmet legal needs through responses to questions about “social assistance needs.”^43^ For example, Luisa Schaeffer, a Patient Navigator at the Brockton Neighborhood Health Center with “deep roots in the community,” was able to restore a COVID-positive patient’s Supplemental Nutrition Assistance Program (SNAP, formerly Food stamps) benefits in just one day by texting a local bureaucrat at the welfare agency.^44^ Importantly, the model provided resource coordinators access to the CTC’s attorney when legal issues were unable to be resolved through informal advocacy.^45^

Medical-Legal Partnership (MLP) is a model for integrating legal services in health care settings that has proven useful in benefitting communities during the pandemic.^46^ Most MLPs draw on a variety of funding sources including their health care organization partners, philanthropy, and government grants.^47^ Sources of public investment in MLPs have increased in recent years, with more states adopting innovative Medicaid financing models that include funding for legal services^48^ and Congressmembers introducing legislation to support MLPs through a new grant program administered by states.^49^ In addition, academic MLPs — those housed in or affiliated with academic institutions — offer unique contributions for advancing health justice, such as catalyzing interprofessional collaborations to benefit communities, advance research, and train learners.^50^ For example, the COVID Equity Response Collaborative Loyola (CERCL) is “a multi-disciplinary collaborative network of academic, community, public, and institutional partners” established by Loyola University Chicago faculty and staff to respond to the health, social, and legal needs of minority communities living in the Chicago suburb of Maywood.^51^ CERCL includes MLPs at the Health Justice Project of Loyola University Chicago School of Law and Legal Aid Chicago.^52^ CERCL has adopted an anti-racist mission of “minimize[ing] the negative impact from COVID-19 in Black and Latinx communities.”^53^ Initially supported by the university exclusively, CERCL obtained a private grant and funding from the Cook County Department of Public Health to expand its work.^54^ Another example of MLPs being integrated into contact tracing programs is the COVID-19 Workers’ Rights Helpline developed by the MLP at California Rural Legal Assistance (CRLA), a legal services organization that primarily serves rural farmworkers.^55^ Legal services organizations are common MLP legal partners that are often funded through a patchwork of grant funding, including from the Legal Services Corporation, Interest on Lawyer’s Trust Accounts Funds, state and local appropriations, foundation grants, *cy pres* awards, and philanthropic donations.^56^ The Monterey County Health Department, which helps to fund the MLP at CRLA, referred COVID-positive farmworkers to the Helpline to address unmet social and legal needs.

Integrating sociolegal needs screening and service provision into well-functioning contact tracing systems during the COVID-19 pandemic may yield lessons that can apply to future surges of the virus and future pandemics, mitigating their impact on population health and health care costs.^57^ By analyzing risk factors for exposure to COVID-19 and barriers to isolation and quarantine that are collected by contact tracers, organizations can more effectively design prevention and mitigation efforts for socioeconomically vulnerable populations.^58^

Although integrating sociolegal needs screening and service provision into contact tracing would provide tangible benefits to communities facing the greatest health risks, it is, by no means, a panacea for the racial, ethnic, and poverty-related health disparities that plague the nation.^59^ Rather, it could be a stopgap to address an urgent need during the COVID-19 pandemic and future outbreaks. It does not provide a sustainable path for addressing the deeper structural inequities that precipitated the crisis.^60^

While scaling up interventions to address unmet social and legal needs for people affected by the COVID-19 pandemic is wise, the best interventions pre-existed the pandemic and will outlast it. For example, New York City Health + Hospitals, the largest public health care system in the country, had a program in place before the COVID-19 pandemic to address needs relating to food insecurity, housing, income support, and legal resources, which could provide a model for integrating them into contact tracing efforts.^61^ Because it had already invested in this system, it responded nimbly to increased patient needs during the pandemic.^62^ In addition, the health system’s administrators have already observed that screening tools, trainings, and resource lists developed to serve COVID-19 patients about to be discharged from the hospital will be valuable for designing holistic services to a broader group of patients after the pandemic.^63^ Most health systems will need to expand their efforts to collect data about patients’ unmet sociolegal needs in order to mitigate negative population health consequences and excessive costs during the next surge or pandemic.^64^

Beyond integrating health care, public health, and human and legal services at the level of the individual patient, the COVID-19 pandemic should inspire joint advocacy and investments at the population level.^65^ Scholars have identified the fragmented structure of the health care, public health, and human services systems in the United States as a barrier to addressing the root causes of poor health.^66^ Health care providers can help public interest advocacy organizations make a compelling case for increased public investment in social services, including “paid sick leave; eviction and utility shut-off moratoriums; temporary housing for homeless individuals and exposed low-income individuals who otherwise may not be able to protect their families; expanded unemployment insurance; economic support for undocumented immigrants; and protections for jail and prison inmates.”^67^ In addition, health care providers who understand the connections between unmet legal needs and poor health can be powerful advocates for increased funding for civil legal aid organizations, particularly those with whom they partner through an MLP.^68^ Existing public investments in civil legal aid, recent innovations in the use of Medicaid dollars to support MLPs, and the urgent need to strengthen our public health infrastructure may allow health justice advocates to imagine a horizon in which legal services are integrated into government-led public health responses, despite the barriers yet to overcome. The results of successful advocacy on these issues will outlast the current pandemic and may help to prevent or curtail the next one.

## Conclusion

Integrating sociolegal needs screening and service provision into contact tracing protocols during an outbreak of infectious disease can help to mitigate negative public health consequences, conserve health care resources, and alleviate outbreak-related health disparities. Although enhancing contact tracing in this way during the COVID-19 pandemic and future pandemics will not address the structural inequities that underly the racial, ethnic, and poverty-related disparities in morbidity and mortality, it may help to herald a more sustainable integration of health care, public health, and human and legal services, which has the potential to have a greater impact.
